# Integrative modeling of the HIV-1 ribonucleoprotein complex

**DOI:** 10.1371/journal.pcbi.1007150

**Published:** 2019-06-13

**Authors:** David S. Goodsell, Andrew Jewett, Arthur J. Olson, Stefano Forli

**Affiliations:** 1 Department of Integrative Structural and Computational Biology, The Scripps Research Institute, La Jolla, California, United States of America; 2 Center for Integrative Proteomics Research, Rutgers State University, Piscataway, New Jersey, United States of America; University of Houston, UNITED STATES

## Abstract

A coarse-grain computational method integrates biophysical and structural data to generate models of HIV-1 genomic RNA, nucleocapsid and integrase condensed into a mature ribonucleoprotein complex. Several hypotheses for the initial structure of the genomic RNA and oligomeric state of integrase are tested. In these models, integrase interaction captures features of the relative distribution of gRNA in the immature virion and increases the size of the RNP globule, and exclusion of nucleocapsid from regions with RNA secondary structure drives an asymmetric placement of the dimerized 5’UTR at the surface of the RNP globule.

## Author summary

The genome of HIV-1 is composed of two strands of RNA that are packaged in the mature virion as a condensed ribonucleoprotein complex with nucleocapsid, integrase, and other proteins. We have generated models of the HIV-1 ribonucleoprotein that integrate experimental results from multiple structural and biophysical experiments, exploring several hypotheses about the state of the RNA before condensation, and the role of crosslinking by integrase. The models suggest that the 5’UTR, which shows extensive secondary structure, has a propensity to be placed on the surface of the condensed globule, due to reduced binding of nucleocapsid to double-stranded regions within the 5’UTR. This unexpected localization of the 5’UTR may have consequences for the subsequent structural transitions that occur during the process of reverse transcription.

## Introduction

**Fig 1:**
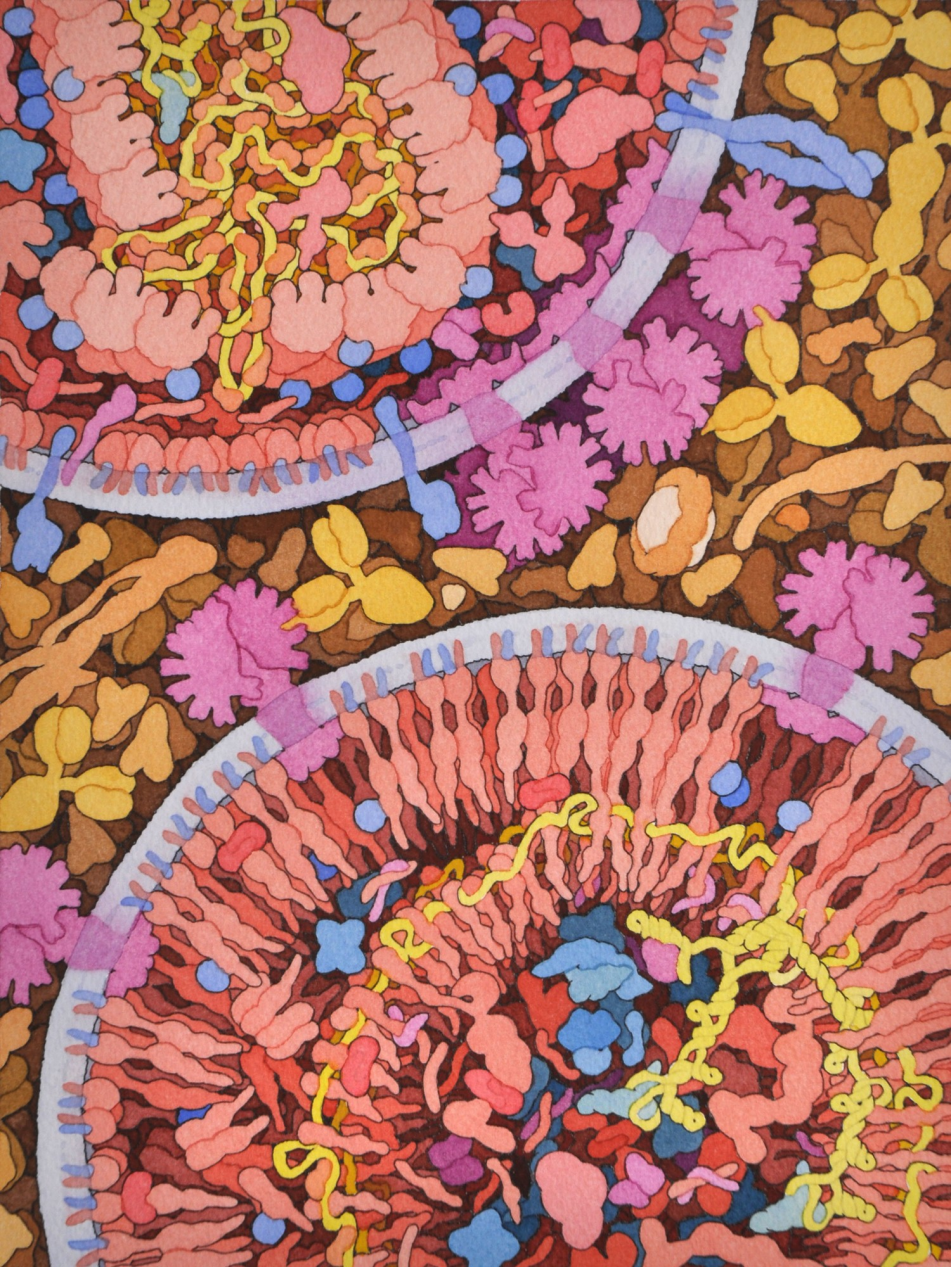
Artistic rendering of the maturation of HIV-1 [3]. An immature virion is shown at the bottom, with HIV-1 protease cleaving the gag and gag-pol polyproteins (red). The viral genome (yellow) is released, condenses with nucleocapsid and integrase, and is encapsidated, as seen in the almost-mature virion at the top. Other molecules in the illustration include: envelope glycoprotein (magenta), host cell proteins (blue), and blood serum proteins (shades of tan between the two virions). Illustration available at the HIVE Center, (http://hive.scripps.edu/resources.html).

Electron microscopy of mature HIV-1 shows a condensed ribonucleoprotein (RNP) complex [1] packaged within the cone-shaped capsid, which is thought to include genomic RNA, nucleocapsid, integrase, transfer RNA, reverse transcriptase, and other components [2]. It is formed through a complex, multistep process where genomic RNA (gRNA) associates with a lattice of Gag polyproteins at the cell surface, which buds from the surface to form an immature virion, followed by proteolytic cleavage of Gag into capsid, nucleocapsid, integrase and other viral proteins, and finally condensation and encapsidation of the mature RNP within the capsid (Fig 1). Understanding of the supramolecular and molecular details of this RNP is important, since it must undergo large structural transitions over the course of the viral life cycle, including playing a central role in assembly of Gag proteins into new viruses and transition from single-stranded RNA to double-stranded DNA during reverse transcription.

The HIV-1 RNP has been studied by many complementary experimental methods. As revealed by SHAPE and other methods, HIV-1 genomic RNA dimerizes and has an extensive secondary structure [4,5], including a well-documented structure at the 5’ untranslated region (5’UTR) [6]. Nucleocapsid coats the gRNA at a density of about 1 nucleocapsid per 11–12 nucleotides and plays key roles as a chaperone in reverse transcription and other processes [7]. A recent study took advantage of the extreme radiation sensitivity of nucleocapsid, which causes formation of bubbles in tomograms, to localize nucleocapsid in the RNP and further show a preference for localization in the dense condensate at the large end of the capsid [8]. Studies on the mode of action of ALLINI compounds (allosteric integrase inhibitors) have revealed that integrase is also essential for maturation of the RNP, and crosslinks the RNA in the mature virion [9].

As part of our ongoing work to study the mesoscale structure of HIV virions at various stages of the viral life cycle, we have developed coarse-grain models of the mature HIV-1 RNP to explore its formation and characteristics, integrating experimental results from electron microscopy, structural biology, CLIP-Seq and SHAPE. Coarse-grain models have been instrumental in understanding of mesoscale-level protein interactions in HIV structure and maturation, including models of the structure of immature virion [10] and formation of the cone-shape capsid [11, 12]. In particular, these studies reveal the process whereby gag protein in immature virions assembles into an imperfect hexagonal lattice. CryoEM studies have further revealed that this hexagonal lattice covers roughly 2/3 of the inner surface of the immature virion, with scattered defects [13]. In the work reported here, we interpret these results with a quasisymmetrical model of gag in immature virions, where the defects correspond to missing gag hexamers at the pentagonal sites in the quasisymmetrical lattice. We then generate models of the mature RNP that explore several alternatives for how the gRNA is deposited onto this immature gag lattice, and how much of this immature virion structure is retained when the RNP matures.

Our RNP models include three elements: two copies of 9 kb gRNA, 2000 nucleocapsid proteins, and 140 integrase subunits. The coarse-grain method begins with several assumptions for the structure of the genome within the immature virion, folds the 5’UTR based on experimentally-determined base-pairing interactions, and condenses the RNA through interaction with nucleocapsid and integrase to form a mature RNP.

## Results

### Summary of the protocol

The coarse-grain modeling method used here builds on previous lattice-based methods for modeling bacterial nucleoids [14]. Briefly, the method begins with an initial geometric model of the gRNA built within a sphere representing the Gag polyprotein lattice in the immature virion, and assigns integrase crosslinking sites based on proximity of the experimentally-determined binding sites. The model is then condensed, driven by the weak attraction of nucleocapsid and gRNA, while being constrained by integrase crosslinks and steric bulk of nucleocapsid. Three initial gRNA configurations were tested, based on three different hypotheses for the capture and maturation of the gRNA. “Self-avoiding Gag” models deposit the two gRNA strands on the inner surface of the immature Gag lattice in a self-avoiding random walk, assuming that integrase crosslinking occurs soon after release of the gRNA. This model explores the hypothesis that local features of the RNA placement on the Gag lattice may be retained as the complex condenses. “Overlapping Gag” models similarly deposit the two gRNA strands on the Gag lattice, but allow them to overlap. “Random” models assume that the RNA is released in the immature virion and is randomly distributed within the available volume before condensation. Integrase is known to adopt multiple oligomeric states [15], so separate models with integrase tetramers and with integrase dimers were tested, as well as models without integrase.

### General features of the RNP

**Fig 2:**
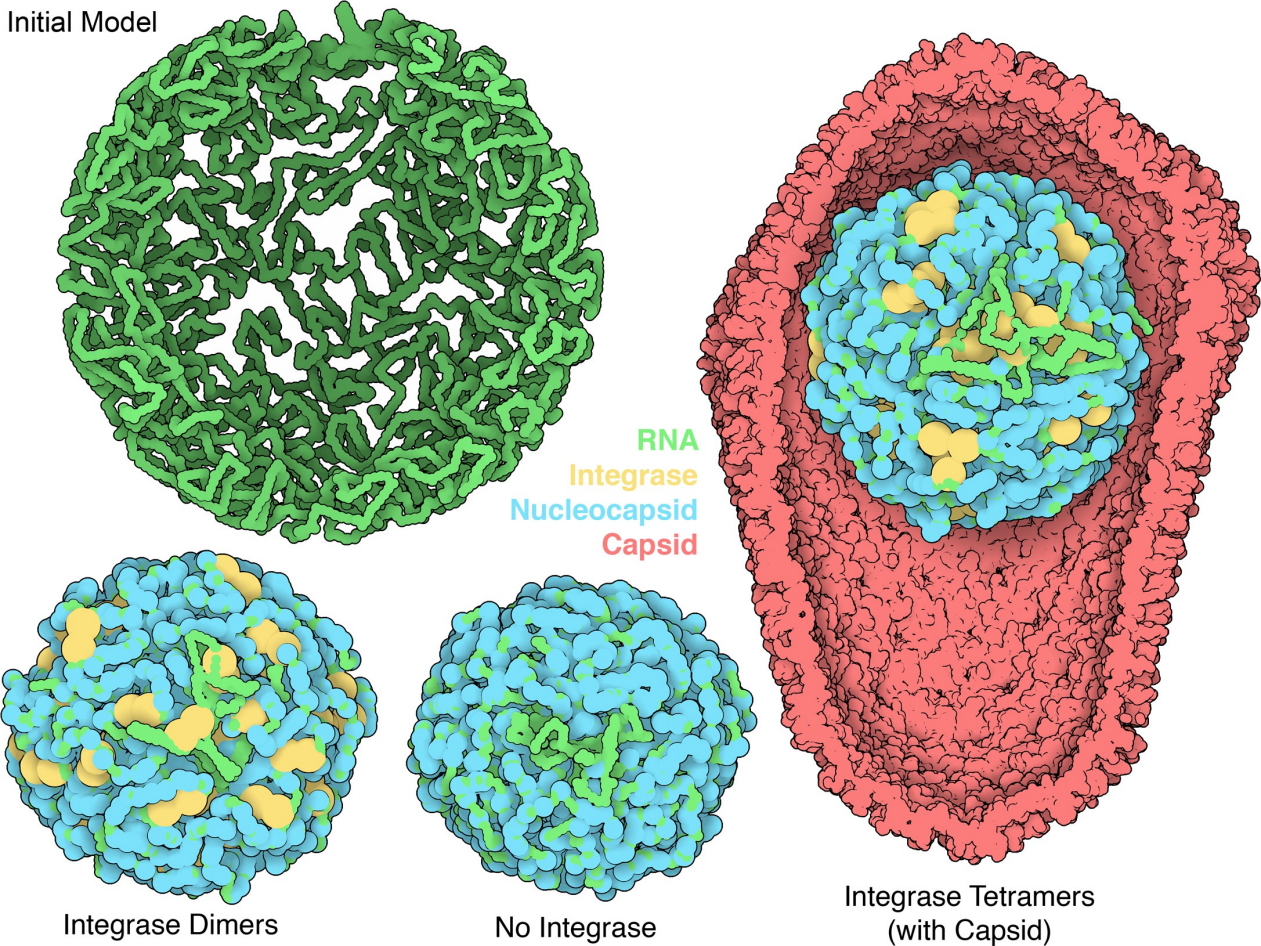
HIV-1 RNP from self-avoiding Gag models. Genomic RNA is shown in green, integrase in yellow and nucleocapsid in blue. The RNP with integrase tetramers is shown in the context of the HIV-1 capsid (PDB entry 3j3q, shown in red).

Coarse-grain models of the mature HIV-1 RNP show a uniform, condensed form (Fig 2). Volumes decrease by roughly a third to a quarter relative to the initial models (Table 1). The three initial configurations (Random, Self-avoiding Gag, and Overlapping Gag) yield similar volumes of the final models. As seen in Fig 2, the RNP globule easily fits within the experimentally-determined structure of the capsid, and matches closely images of the intact capsid from electron microscopy (see, for example, [1]).

**Table 1:** Volume of nucleoid models.

	Volumes in nm^3^ (standard deviation)			
RNA configuration	initial model	minimized (IN tetramers) (1)	minimized (IN dimers)	minimized (no IN)
Self-avoiding Gag	180300 (7500)	70500 (2200) 39.1%	61900 (2200) 34.3%	52300 (1500) 29.0%
Overlapping Gag	182300 (4700)	70400 (970) 38.6%	60900 (2300) 33.4%	51800 (14002) 28.4%
Random	202000 (3400)	70300 (1600) 34.8%	61200 (1400) 30.3%	51500 (2300) 25.5%
	**140 IN subunits**	**80 IN subunits**	**40 IN subunits**	**No IN**
Self-avoiding Gag, IN tetramer	70500 (2200)	63700 (2100)	58100 (1600)	52300 (1500)
Self-avoiding Gag, IN dimer	61900 (2200)	58200 (1800)	55500 (1700)	52300 (1500)
	**Square Planar**	**Square Planar, Exchanging**	**Tetrahedral**	**Tetrahedral, Exchanging**
Self-avoiding Gag, IN tetramer	70500 (2200)	69800 (4600)	61200 (2000)	60800 (1900)

(1) for miniminized models, volume percentages with respect to the initial models are reported

Models with integrase tetramers showed the greatest volume, followed by globules with integrase dimers, and models with no integrase were smallest. Control experiments varying the number of dimers and tetramers, and comparing the default square-planar model of integrase with a tetrahedral model, show that the difference in size is a direct consequence of the volume occupied by the integrase (Table 1). For example, the default square planar model for the integrase tetramer includes two steric spheres with 9.2 nm diameter that may overlap, so the total volume of 35 integrase tetramers would be in the range of 14300 to 28500 nm^3^. In the Self-avoiding Gag model, adding these values to the model with no integrase gives a range of 66600–80800 nm^3^, which is consistent with the model with integrase tetramer at 70500 nm^3^. As a control, we also created models using tetrahedral integrase tetramers composed of two dimers, each using the same representation used for the dimer models (6.5 nm diameter spheres). The tetrahedral tetramer model shows a volume of 61200 nm^3^, similar to the model with integrase dimers at 61900 nm^3^. Finally, we did an experiment that would be consistent with rapid exchange of nucleocapsid, by creating the condensed model of RNP without NC, and then choosing IN positions in the condensed globule. The resultant globule (69800 nm^3^) showed a similar volume as the globule with integrase crosslinking throughout condensation (70500 nm^3^).

**Fig 3:**
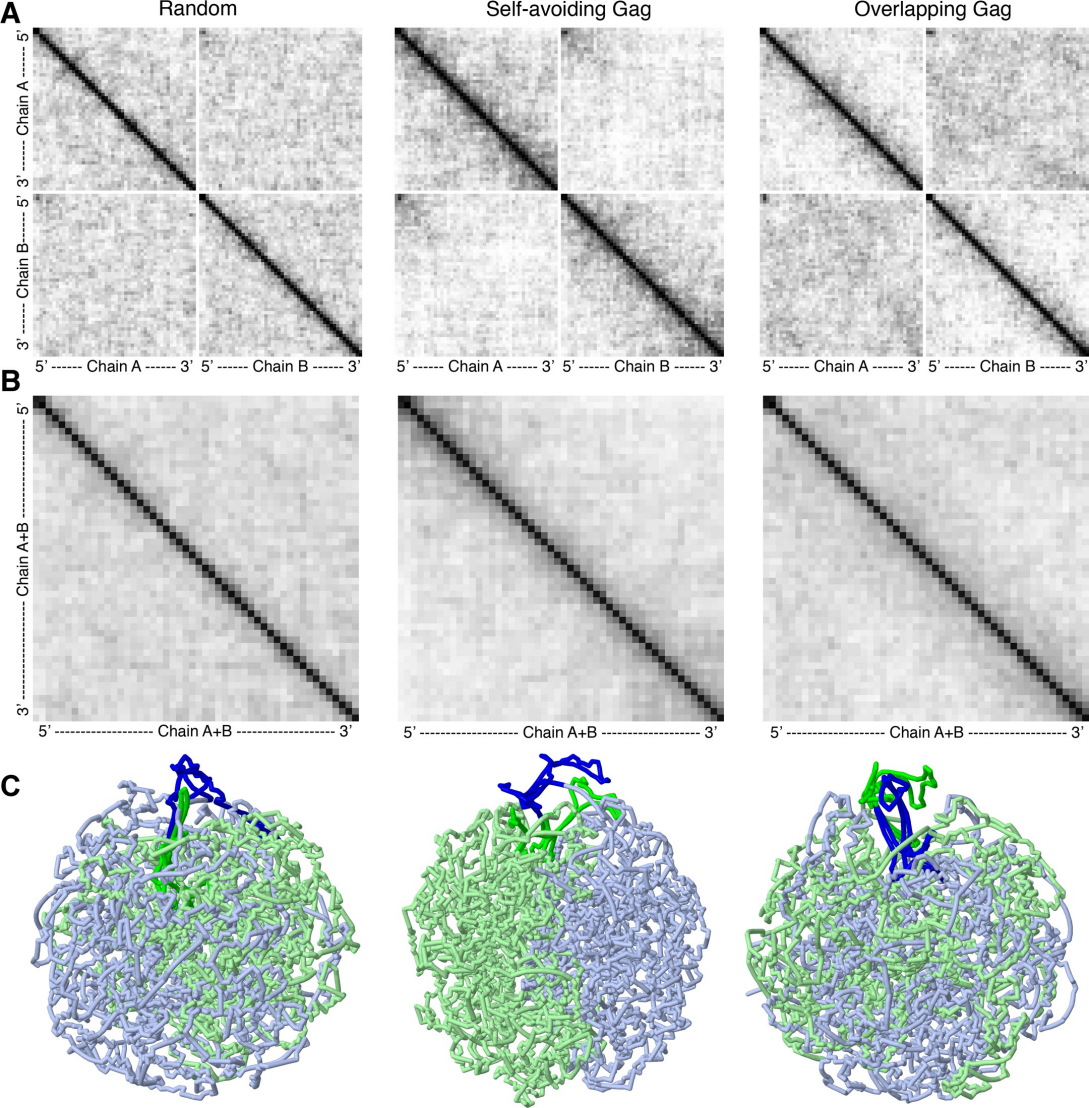
Contact probability plots for three models with integrase tetramers. (**A**) Plots include data treating the two chains individually. (**B**) Plots combining intra-chain and inter-chain contacts. The 5’UTR of each chain is in the two bins at upper left of each plot. (**C**) Representative models from sets with IN tetramers. Only the gRNA is shown, with the 5’UTR aligned at the top.

**Fig 4:**
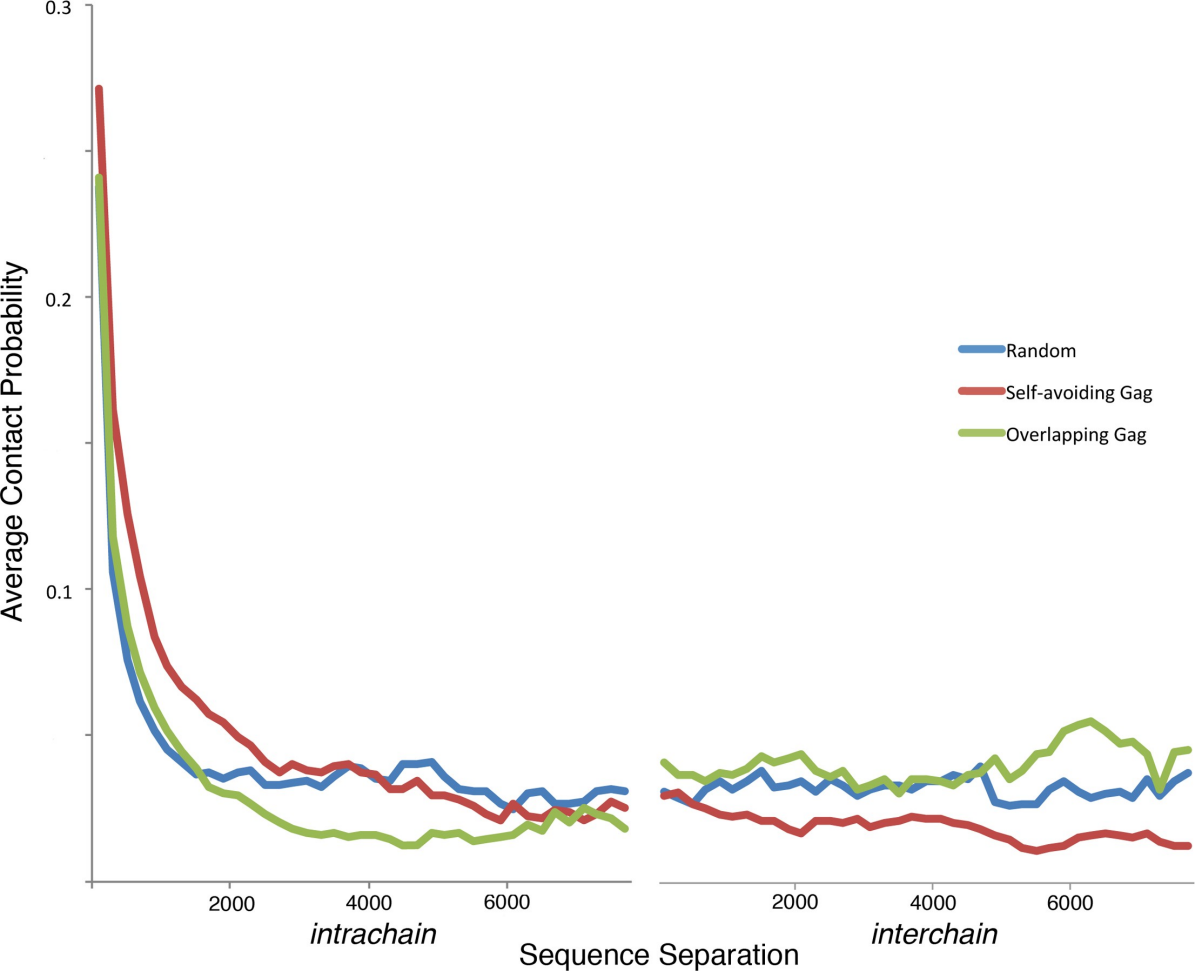
Average contact probability for a range of separations of nucleotides within each chain and between the two chains.

Contact probability plots (Fig 3) reveal subtle differences between the three types of models, and these are further quantified by plotting the average value of the contact probability as a function of the separation of nucleotides within or between chains (Fig 4). Models built from the Random configurations show a random sprinkling of interactions between all regions of the gRNA, and a similar distribution of contacts between the two chains. Regions immediately adjacent to the diagonal are sparsely populated, showing the lack of organization at small scales, such as plectonemic supercoils or hairpins. Conversely, models with the Self-avoiding Gag configuration show more interaction near the diagonal. This is expected, since the contact probability of a random chain decays less rapidly with loop length when constrained in 2D compared with 3D [16]. Cross-chain interactions, on the other hand, are reduced due to the self-avoiding definition of the model. Models starting from the Overlapping Gag configurations show a reversed nature: the interaction along the diagonal is slightly reduced when compared to models starting with the Self-avoiding Gag configuration, but the cross-chain interactions are enhanced, presumably due to the many points of close proximity between chains in the initial model.

As expected, all three models show a strong interaction at the 5’UTR, due to the modeled secondary structure. A subtle band of reduced interaction extends horizontally and vertically from the diagonal at the 5’UTR, indicating that the 5’UTR forms fewer than expected interactions with the rest of the chains. As shown below, this is a consequence of a general exclusion of the 5’UTR from the body of the globule.

We also calculated contact probability plots that are averaged over the two strands, which are more indicative of results that may be obtained from a hypothetical high-resolution contact experiment (Fig 3B). The underrepresented band extending from the 5’UTR is distinguishable, but the more global features that differ between the three models are not as distinguishable between the three plots.

### Exclusion of the 5’UTR

**Fig 5:**
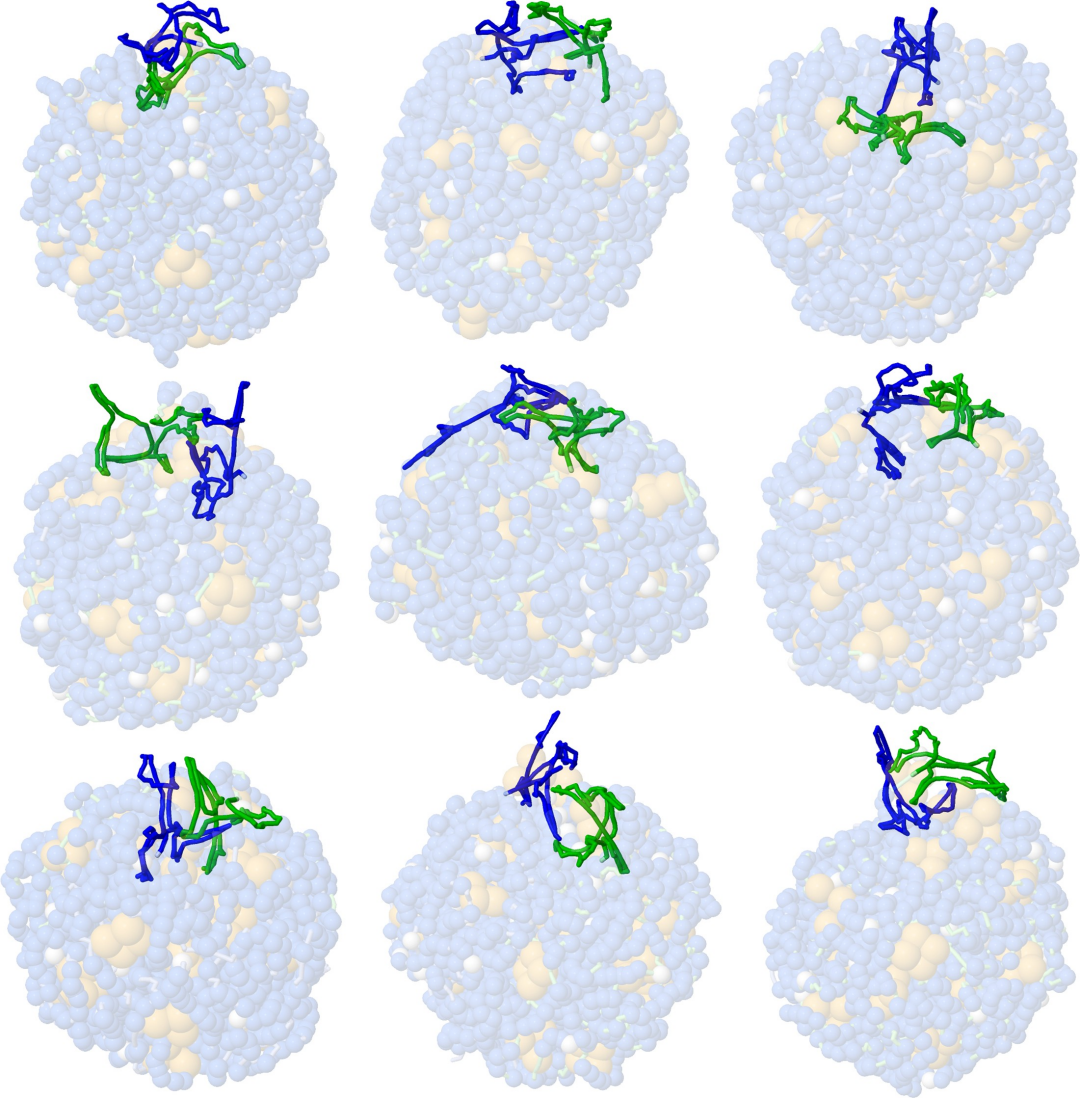
Exclusion of the 5’UTR from the RNP globule. The first nine models from the Self-avoiding Gag starting model with tetramers. The 5’UTR is shown in blue and green, and the rest of the model is transparent.

Given the coarse-grain nature of the model, the hairpin loops in the 5’UTR do not show a typical double helical structure, but rather end up looking more like distorted bobby pins (see the examples in Fig 5). We noticed early in this study that the secondary structure has a consequence on the global nature of the RNP: during the process of condensation, the 5’UTR is excluded from the body of the globule and often ends up on the surface. We quantified this exclusion with a simple metric, by evaluating the average radial distance of nucleotides in the 5’UTR as compared with the rest of the gRNA and the sites of integrase interaction (Table 2). The central column of this table shows that the average radius of the bulk of the RNA is fairly constant across the default model and several controls, with models with IN tetramers at just over 18 nm, and models with IN dimers or no IN slightly smaller. The right column shows that the IN-binding sites show a similar trend across the default and control models. The 5’UTR shows different behavior, however, based on the assumptions made for the nature of region.

**Table 2:** Average radii of RNA regions in RNP models.

		Average Radius (std. dev.) nm (2)		
RNA (1)	integrase	5'UTR	Non-5’UTR	IN sites
**Self-avoiding Gag**				
FullModel	tetramer	20.45 (3.17)	18.40 (0.27)	18.35 (0.20)
FullModel	dimer	20.30 (2.56)	17.33 (0.21)	17.59 (0.24
FullModel	none	18.01 (2.86)	16.69 (0.16)	17.09 (0.23)
TwoChain	tetramer	23.06 (2.27)	18.33 (0.22)	17.29 (0.19)
TwoChain	dimer	22.73 (1.94)	17.35 (0.17)	17.76 (0.23)
TwoChain	none	22.28 (1.14)	16.58 (0.11)	17.29 (0.19)
FullAllNC	tetramer	14.60 (2.84)	18.53 (0.34)	16.90 (0.48)
FullAllNC	dimer	11.97 (2.13)	17.57 (0.34)	17.48 (0.27)
FullAllNC	none	10.11 (2.69)	16.81 (0.51)	16.90 (0.48)
TwoAllNC	tetramer	17.46 (2.95)	18.55 (0.24)	18.38 (0.23)
TwoAllNC	dimer	17.16 (2.11)	17.31 (0.38)	17.58 (0.39)
TwoAllNC	none	16.21 (2.50)	16.62 (0.32)	17.03 (0.27)
**Overlapping Gag**				
FullModel	tetramer	18.93 (3.21)	18.41 (0.33)	18.24 (0.31)
FullModel	dimer	19.36 (2.96)	17.39 (0.19)	17.54 (0.26)
FullModel	none	18.30 (2.63)	16.62 (0.21)	17.04 (0.20)
TwoChain	tetramer	20.84 (2.48)	18.53 (0.19)	18.18 (0.19)
TwoChain	dimer	21.53 (1.82)	17.40 (0.27)	17.16 (0.22)
TwoChain	none	21.72 (1.40)	16.51 (0.22)	17.15 (0.26)
FullAllNC	tetramer	12.34 (2.35)	18.82 (0.17)	18.03 (0.19)
FullAllNC	dimer	11.22 (2.24)	17.77 (0.16)	16.99 (0.22)
FullAllNC	none	9.19 (2.00)	16.89 (0.26)	16.88 (0.32)
TwoAllNC	tetramer	17.16 (2.57)	18.68 (0.15)	18.16 (0.23)
TwoAllNC	dimer	16.85 (1.80)	17.55 (0.22)	17.08 (0.23)
TwoAllNC	none	16.17 (2.18)	16.42 (0.70)	16.96 (0.64)
**Random**				
FullModel	tetramer	20.41 (4.83)	18.41 (0.32)	18.30 (0.26)
FullModel	dimer	20.03 (3.41)	17.31 (0.41)	17.62 (0.34)
FullModel	none	17.24 (3.69)	16.63 (0.30)	17.04 (0.27)
TwoChain	tetramer	19.57 (2.25)	18.60 (0.20)	17.93 (0.20)
TwoChain	dimer	20.41 (2.40)	17.59 (0.23)	16.94 (0.24)
TwoChain	none	19.82 (2.25)	16.47 (0.35)	17.06 (0.31)
FullAllNC	tetramer	13.09 (3.07)	18.84 (0.16)	18.07 (0.20)
FullAllNC	dimer	11.93 (2.47)	17.68 (0.16)	16.98 (0.18)
FullAllNC	none	10.00 (2.54)	16.78 (0.42)	16.91 (0.42)
TwoAllNC	tetramer	17.48 (2.54)	18.67 (0.29)	18.07 (0.25)
TwoAllNC	dimer	17.49 (1.45)	17.55 (0.36)	17.15 (0.32)
TwoAllNC	none	15.56 (2.14)	16.31 (1.49)	16.77 (1.37)

(1) “FullModel” models include secondary structure and no random NC positions in the 5’UTR, “FullAllNC” models include secondary structure and NC positions in the 5’UTR, “TwoChain” models treat the gRNA as two disconnected chains with no secondary structure and no NC positions in the 5’UTR, “TwoAllNC” models have two disconnected chains and NC positions along the entire chain.

(2) Average radii are measured for: “5’UTR,” beads in the 5’UTR; “Non-5’UTR,” beads not in the 5’UTR; “IN sites,” beads involved in integrase crosslinks.

Several control experiments help to identify the cause of the exclusion. We tested two hypotheses: the role of NC, and the role of the secondary structure itself. In the default experiments (“FullModel” in Table 2), NC is excluded from the 5’UTR (apart from one site determined experimentally), providing less attractive force for the condensation, resulting in eccentric location of the 5’UTR and larger radial average values. When hypothetical models are condensed with NC covering the 5’UTR as well as the rest of the gRNA (“FullAllNC” in Table 2), the 5’UTR instead is located in the interior of the globule, showing smaller radial average values. This is expected, since the local concentration of nucleocapsid is higher due to the close proximity of the RNA chains when they base pair.

When the two RNA chains are treated as separate chains, with no secondary structure, they show a similar behavior with respect to NC. If NC is excluded from the 5’UTR (“TwoChain” in Table 2), the 5’UTR tends to pack on the surface of the globule, and if NC is equally distributed (“TwoAllNC” in Table 2), the 5’UTR has the same properties as the rest of the chain. From these experiments, we conclude that NC is the primary cause of the exclusion of the 5’UTR under the assumptions made by our modelling method.

## Discussion

One of the major goals of this work is to create plausible models of the RNP to identify experimental modalities that could distinguish between different hypotheses for the effect of integrase crosslinking on the final form of the RNP. Note that our protocol is not meant to simulate the process of condensation, rather, it is designed to provide a rapid method for generating multiple models that are consistent with the available data defining the nature of the RNP. The three types of models tested here (Self-avoiding Gag, Overlapping Gag, and Random) are designed to explore different hypotheses for the initial structure of the gRNA and the possibility that IN crosslinking could trap features of this structure in the condensed RNP. Contact probably plots reveal differences in the arrangement of chains in the mature RNP, with the self-avoiding model showing stronger partitioning of the two chains in different regions of the RNP. The study also reveals that, because the two gRNA chains are identical, these differences would be difficult to quantify in a hypothetical Hi-C type experiment (Fig 3B).

Two additional morphological features are revealed in these models, which may be accessible to study by cryoEM or super-resolution microscopy. First, crosslinking by integrase leads to a larger condensed globule. Control experiments revealed that this increase in size is primarily due to the steric bulk of the incorporated integrase subunits. However, the model for integrase used here is very simple, with no constraints on the relative orientation of the gRNA strands that are bound. We might expect that the condensed globule may be larger if integrase is more rigid than modeled here, with reduced mobility in the linker to the RNA-binding C-terminal domain and consequently stronger constraints on the orientation of the gRNA binding sites. Unfortunately, there currently are no convincing models of integrase structure at this stage in viral life cycle, but based on available structures of integrase dimers and intasomes (see Methods), we might expect that the connection to the C-terminal domains is quite flexible. Our control experiments suggest that we would not expect a smaller condensed globule if integrase is found to exchange sites rapidly during the process of condensation.

The second emergent feature of the models is the exclusion of the 5’UTR from the bulk of the RNP globule. Control experiments implicate the binding propensity of NC for single-stranded nucleic acids [17] as the cause of this exclusion. Exposure of the 5’UTR might be expected to have functional consequences, for example, by allowing ready access to reverse transcriptase for the initiation of genomic DNA synthesis.

The current model includes only gRNA, nucleocapsid and integrase, and secondary structure only in the 5’UTR. There are many opportunities for future studies to explore additional functional features and their emergent effects on RNP globule structure and partitioning. These will include a more detailed study of secondary structure, starting first with the Rev response element and extending to more detailed models as defined by SHAPE data. In addition, other molecules, such as transfer RNA and reverse transcriptase, are known to interact with the gRNA and could have effects on the form and function of the RNP. We are also currently developing methods for generating full atomic models from these coarse-grain representations, for use both in simulation and educational outreach.

## Materials and methods

### Generation of initial models

#### Random model

For the Random initial configuration of the gRNA, we performed a short simulation of a self-avoiding worm-like chain [18] to give a well-mixed configuration that fills the available space in the immature virion. The models were prepared using MOLTEMPLATE (moltemplate.org) and run using Langevin dynamics in LAMMPS [19]. A dimer of gRNA, with the two strands connected at their 5’ ends, was modeled as a single chain of 6116 beads (3 bases/bead) bounded by a 38 nm radius sphere representing the free space within a typical immature virion. Strong springs were used to constrain neighboring beads, but 3-body angular interactions and higher were ignored given the low persistence length of single-stranded nucleotide chains [20]. The resulting persistence length of the simulated polymer was 2 nm. Nonbonded interactions were modeled using a repulsive Gaussian potential. Simulations were initiated in an arbitrary conformation and nonbonded interactions set to zero to allow the chain to pass through itself and adopt a random shape. The nonbonded barrier strength was then increased stepwise to a final value of 50 k_B_T over the full simulation of 4 million steps. The barrier initially begins at 0.5 k_B_T and doubles every 500000 timesteps, capping at 50 k_B_T. Langevin dynamics was used with a timestep of 0.01τ_m_ and a damping time τ_D_ = 2000τ_m_, where τ_m_ = sqrt(mb^2^/k_B_T), bond length b = 1 nm, and k_B_T and the mass m were arbitrarily set to 1 (the damping time is related to the viscosity (γ) as τ_D_ = m/γ). Note that in the current study, the values of τ_m_, τ_D_, m, and k_B_T are not physically relevant, but were chosen to maximize the randomness of the resulting conformation while keeping the simulation simple and the running time short.

#### Gag models

**Fig 6:**
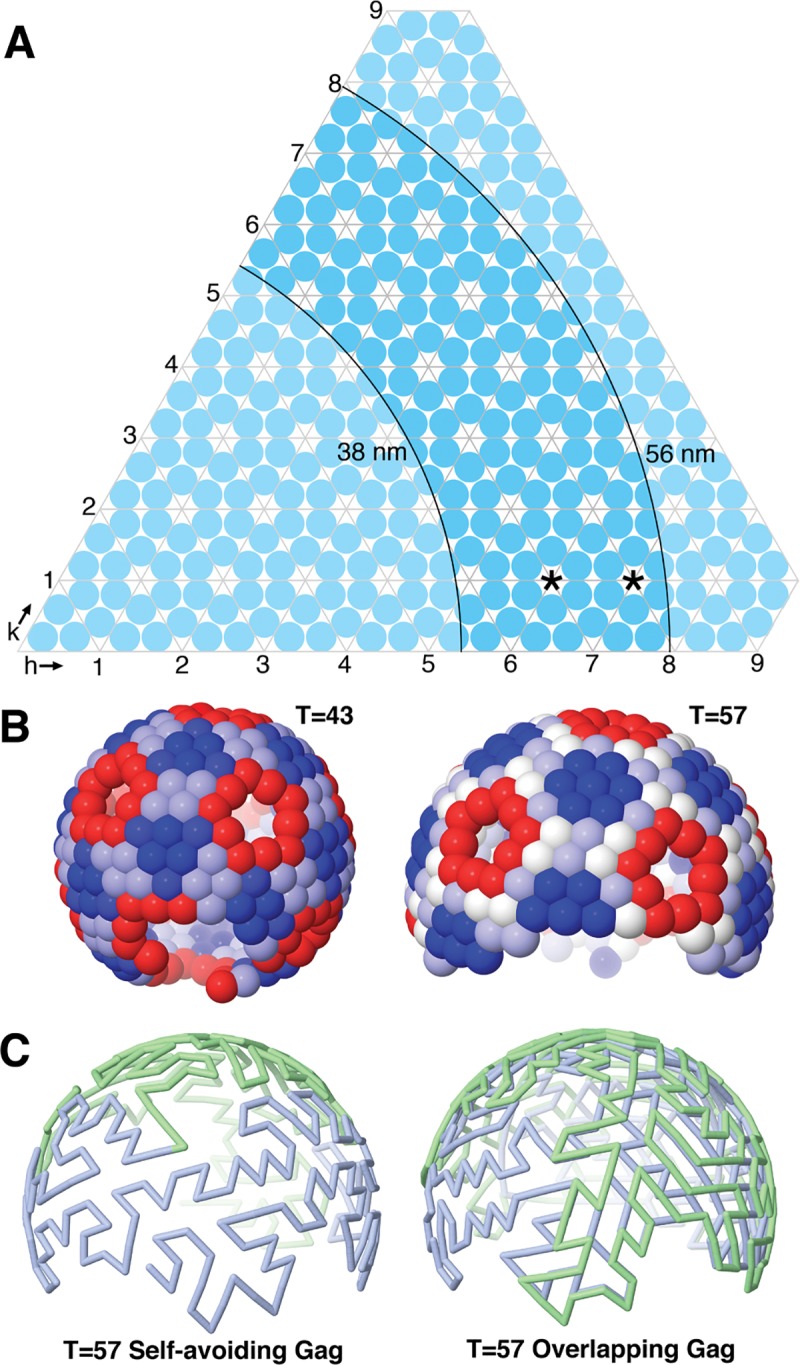
Quasisymmetrical Gag models. (**A**) Quasisymmetrical T-number diagram (adapted from http://pdb101.rcsb.org/learn/paper-models/quasisymmetry-in-icosahedral-viruses). T-numbers that create Gag polyprotein lattices that are consistent with cryoEM studies are found between the two arcs. (**B**) Two models of Gag packing. Each sphere represents a Gag hexamer, colored based on deviations from ideal hexameric packing, with red being slightly compressed and blue being slightly expanded. (**C**) Two initial gRNA random-walk paths, with the gRNA strands in blue and green.

The Gag-based initial configurations were created by simulating the lattice of Gag polyprotein in immature virions, then tracing the path of gRNA as it interacts with nucleocapsid domains at each Gag position (Fig 6). Cryoelectron microscopy has shown that hexamers of Gag are arranged in an imperfect quasisymmetrical lattice [21], with hexagonally-packed regions surrounding defects. We modeled quasisymmetric Gag assembly based on these observations from cryoelectron microscopy, assuming that the defects correspond to pentameric sites in the quasisymmetrical lattice. Gag proteins contain multiple domains arranged approximately radially inward from the HIV-1 membrane. Measured at the center of the capsid domains, Gag hexamers have a center-to-center packing distance of 8 nm [13] and a radius of 47 ±9 nm [22].

Quasisymmetrical icosahedra were generated for different triangulations, and vertices of the triangles were projected on a sphere midway between the inscribed and circumscribed spheres of the icosahedron. Triangulations that produced lattices within the observed range of radii were retained. Then, the desired number of hexamers were chosen from the quasisymmetrical lattice by choosing one position at random, then doing a flood fill of neighbors, omitting points near the pentamers since they were distorted from the ideal packing distance. A T = 43 lattice (radius = 46.2 nm) and T = 57 lattice (radius = 53.2 nm) were chosen for further study, and showed similar results in initial tests. The T = 57 lattice was used for the results shown here, since it showed the cup-shaped asymmetry seen in cryoEM, whereas the smaller T-numbers were more spherical.

Positions for nucleocapsid were then generated from this lattice by reducing the radius inwards to 38 nm, the level of nucleocapsid within Gag [22]. Genomic RNA strands were modeled by picking a point at random from the Gag lattice, and generating a self-avoiding random walk that visited any particular point only once. For the Self-avoiding Gag configuration, the random walk was seeded at the site of dimerization of the two strands, and the two strands were grown simultaneously from that point, such that each strand avoided itself and the other strand. A simple random walk was only rarely able to generate a path of the desired length, since it ran into dead ends. The full length was then built using a subdivision approach that picks random neighboring free points along the path and replaces the existing connection with two neighboring connections. For the Overlapping Gag configuration, the two strands were seeded at the same point, but were grown separately, so that each strand avoided itself, but did not see the other strand; one strand was then given a slightly reduced radius. Finally, beads were placed evenly along the segments of these random walks to yield a 3 bp/bead model, which was relaxed and regularized using the positional-relaxation software described below, including only the bonded constraints, hard-sphere repulsion, and a weak repulsive potential.

#### Generation of condensed RNP models

Condensed RNP models were generated using the rapid positional-relaxation method developed from modeling of bacterial nucleoids [14]. As mentioned in the discussion, this method is not designed to simulate the process of condensation in a physically-meaningful manner. Rather, it is designed to create a series of plausible models that are consistent with the assumptions for initial models and the experimental constraints, with modest computational cost. The method begins with a starting model (typically from a lattice-based generation method), and small positional displacement is applied to each bead at each time step, based on a variety of constraints. In the current work, we included bond constraints between neighboring beads, angle constraints based on the distance between beads separated by one bead, hard-sphere steric constraints, a weak attractive constraint, and user-defined crosslinking constraints for secondary structure and IN. The magnitude of displacements and allowable ranges are given in Table 3; the displacements have constant magnitude throughout the protocol and are applied if pairs of beads are outside the allowable ranges. The details of each constraint are included below in the sections on RNA, integrase and nucleocapsid.

**Table 3:** Constraint parameters.

Parameter	Displacement (nm)	Starting Allowable Range (nm)	Ending Allowable Range (nm)
Bonds	0.03	1.0 ±0.05	1.0 ±0.01
Angles	0.004	>1.0	>0.6
Constraints (1)	0.05	D_const_ ±0.05	D_const_ ±0.002
Steric Clash (2)	0.004	>R_a_ + R_b_	>R_a_ + R_b_
Attraction	0.005		
Random (3)	0.04–0.002		

D_const_: constraint distance

R_a_, R_b_: hard-sphere radii of beads a and b

Displacement values are given for the start and end of the protocol

A random displacement is also applied to keep the model from becoming trapped in local minima. At each time step, 2000 random positions are chosen throughout the two chains, and a displacement in a random direction normal to the local bond vector is calculated. This displacement is applied to the position and surrounding area, with the displacement magnitude falling linearly to zero at ±4 beads from the central position. The magnitude of the random displacement is gradually reduced throughout the protocol, akin to reduction of temperature in traditional simulated annealing experiments.

100,000 steps were used for each model generation. Longer protocols did not change the results of our study, but protocols with ~20,000 steps or less often froze into inconsistent conformations. For each type of model, 25 independent instances were created, and all protocols were bounded by a 38 nm radius sphere.

### Definition of the coarse-grain model

#### RNA

RNA was modeled as a flexible worm-like chain with 3 nucleotides per bead. Allowable distances between beads gradually narrowed from 1 nm ±0.05 to 1 nm ±0.01 nm over the course of the protocol. Angles between three successive beads were similarly regularized, disallowing sharply acute angles of less than 60 deg. A hard sphere radius of 1.5 nm was used throughout.

**Fig 7:**
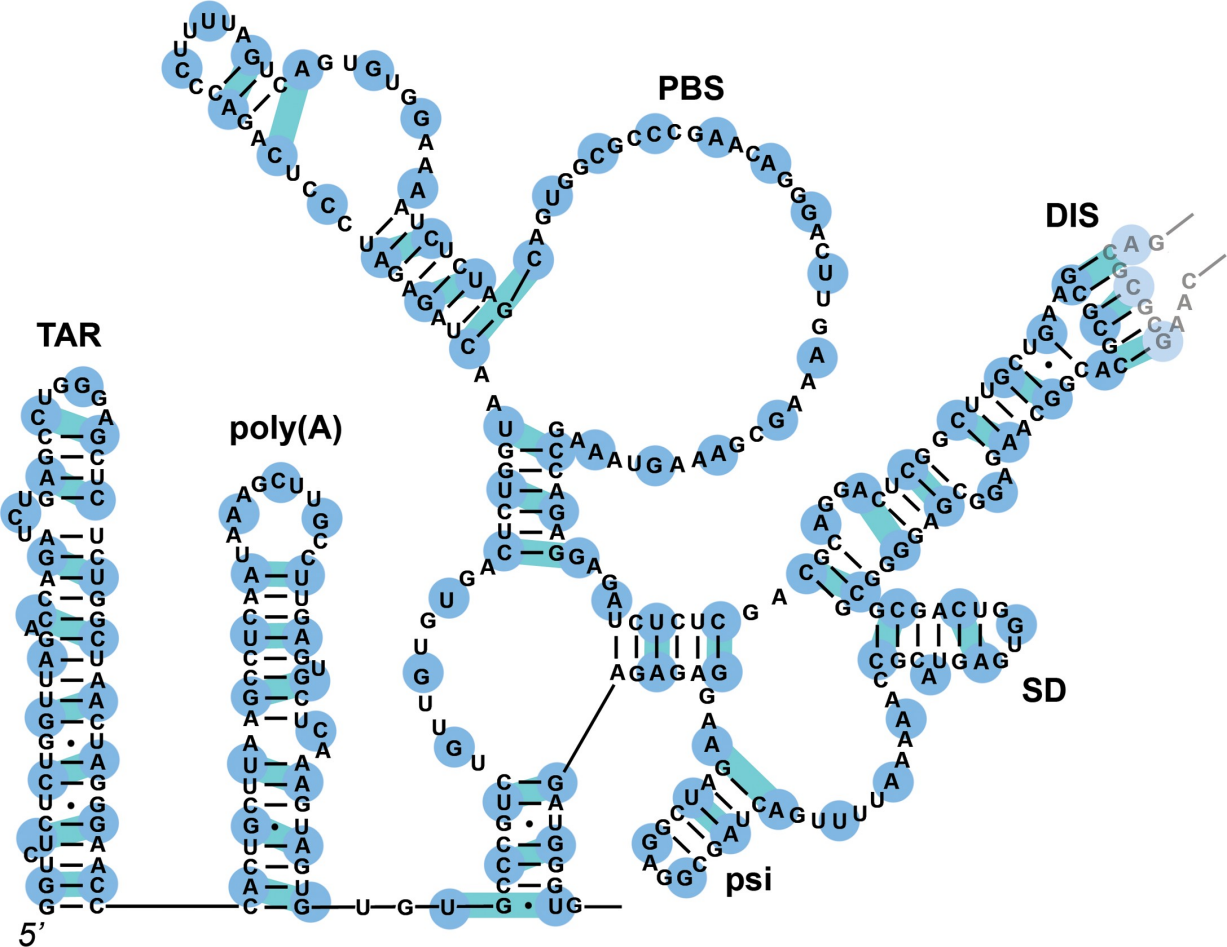
Locations of beads and constraints for the 5’UTR double-stranded regions. Beads for the genomic RNA (blue) represent three nucleotides.

RNA secondary structure was assigned for the 5’UTR region based on SHAPE data, with the rest of the gRNA treated as unstructured. Pairwise constraints that link based-paired regions were assigned manually based on nucleotides 1–341 in Fig 4 of [4], which includes secondary structure for TAR, poly(A) and other hairpins, and the dimerization site (Fig 7).

#### Integrase

**Fig 8:**
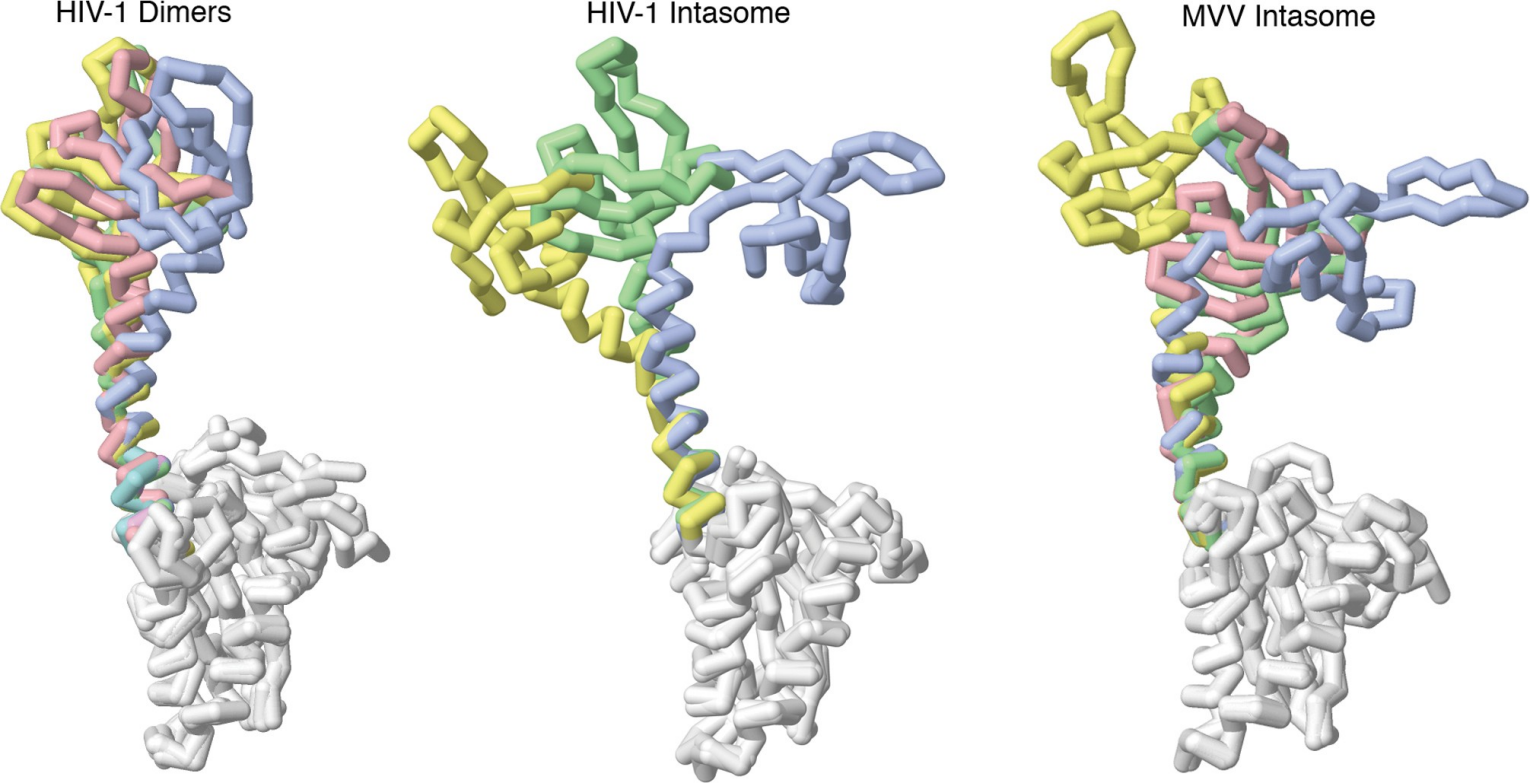
Atomic structures of integrase subunits superimposed at the catalytic domain (white), showing a wide range of flexibility in the C-terminal domains. For HIV dimers, PDB entry 1ex4 is in blue and green and 5hot is in red and yellow. HIV-1 intasome coordinates were kindly provided by Dmitry Lyumkis. MVV intasome is from PDB entry 5m0r.

A variety of experimental observations informed development of the model for integrase. A cluster of basic amino acids in the C-terminal domain of integrase has been implicated as the site of interaction with RNA [9]. The flexibility of the connection between this C-terminal domain and the catalytic domain remains an area of study, so we compared the available structures of HIV-1 and related integrases. By overlapping the catalytic domains of the subunits from these structures (Fig 8), we see large configurational heterogeneity, with the C-terminal domain in several very different positions. The first structure of the full integrase dimer (PDB entry 1ex4) has the C-terminal domain connected to the catalytic domain with an alpha-helical linker. The linker in the “A” chain has a kink, resulting in a roughly 90 degree rotation of the C-terminal domain when compared to the “B” chain. The ALLINI-aggregated integrase dimer (PDB entry 5hot), molecules are locked together into a two-dimensional lattice by strong interactions between the C-terminal domains and the catalytic domains of neighboring molecules, resulting in a conformation similar to the “B” chain of the previous structure. In the structure of a high-order HIV-1 intasome [23] and the similar intasome from lentivirus MVV (PDB entry 5m0r), the C-terminal domains are also linked through an alpha helix, but a wide range of conformations are observed, with bends in the helix, flexibility in the connection between the alpha helix and the C-terminal domain, and complete disorder in one case. Finally, in the structure of a tetrameric HIV-1 intasome (PDB entry 5u1c), the C-terminal domains adopt very different positions relative to the catalytic domain, but no linker was resolved in the cryoEM structure (not shown in Fig 8). Finally, the oligomerization state of integrase in the RNP, and at other stages in the viral life cycle, remains an area of conjecture, so we tested dimers and tetramers in our study.

**Fig 9:**
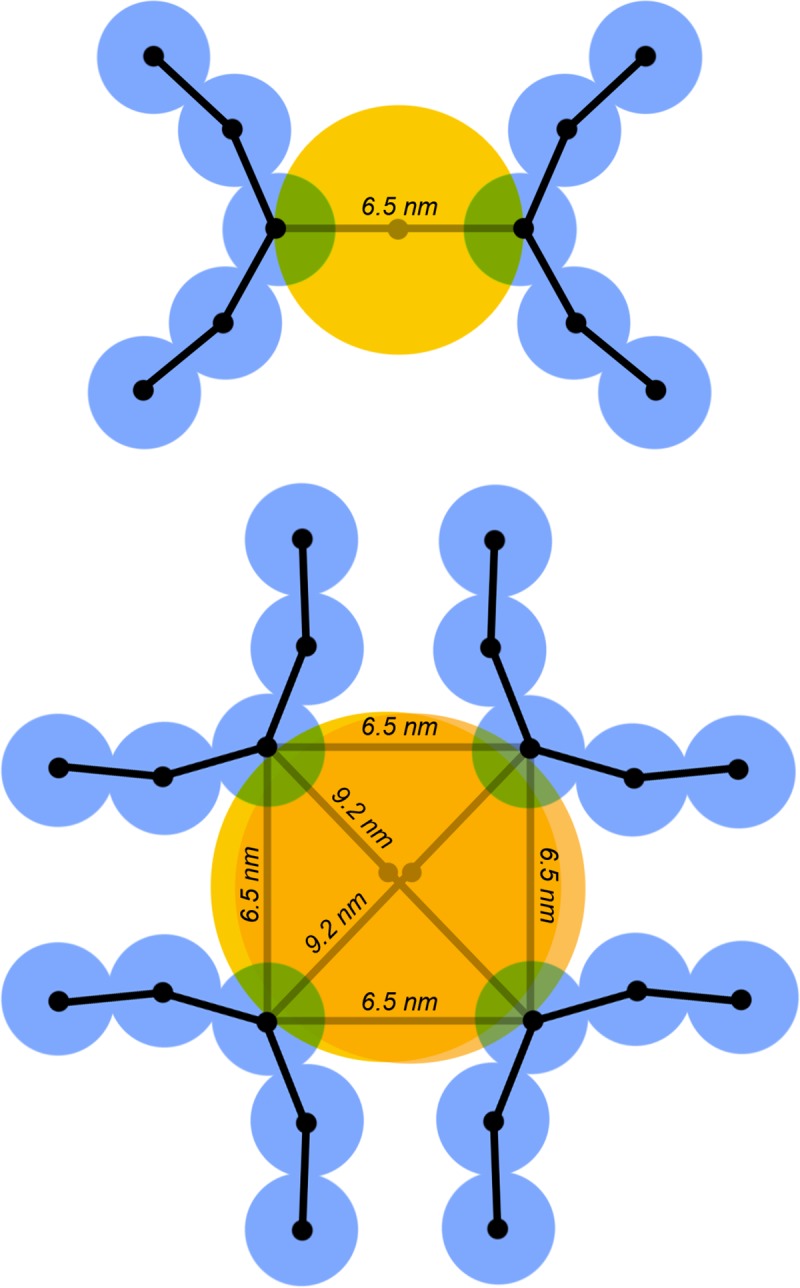
Integrase constraints. The integrase dimer is represented by a single constraint between beads and a steric sphere centered on the constraint (yellow). The integrase tetramer is represented by a square-planar collection of constraints, and steric spheres (which do not repel one another) on the diagonals. A tetrahedral integrase was also tested, with six equal-length constraints and two 6.5 nm spheres.

Based on these observations, we chose a very simple coarse-grain model that does not make specific assumptions about the relative orientations of the C-terminal domains in integrase oligomers (Fig 9). For dimers, we constrain the distance between the two RNA sites to a distance of 6.5 nm, but ignore the geometry of the chains relative to one another. For tetramers, we assume an approximately square planar conformation, and constrain the two diagonals to 9.2 nm and the four edges to 6.5 nm. A 6.5 nm diameter steric sphere is modeled at the center point of the dimer constraint and two overlapping 9.2 nm diameter spheres at the centers of the tetramer diagonal constraints, to provide steric exclusion for non-bonded interactions with surrounding integrase and RNA beads. We also tested a tetrahedral model for the integrase tetramer, with equal constraints of 6.5 nm on all edges of the tetrahedron, and two steric spheres with 6.5 nm diameter on two opposite edges of the tetrahedron. Finally, for visualization, each large sphere is replaced by two smaller beads equally spaced along the constraint(s) to represent the dimer/tetramer.

Binding sites for integrase were taken from CLIP-Seq studies [9]. Peaks were extracted with a scanning window of ±50 nucleotides, searching for points that showed a maximum value across the entire window, yielding 80 sites. These sites were associated with dimers or tetramers based on proximity within the initial coarse-grain gRNA models as follows. A site was chosen randomly, and 1 or 3 neighbors were chosen randomly from the set of neighbors within a given distance threshold, set to 15 nm in the current study. This step was repeated until the desired number of integrase complexes was achieved. Often, the process needed to be repeated multiple times to assign the desired number of integrase crosslinks, given that the number of sites is only slightly larger than the number of integrase subunits.

Estimates for the number of integrase and nucleocapsid vary widely in the literature, with gag estimated at, for example, 1400 [24] to 5000 [25] per virion, and gag:gag-pol ratios from 20:1 [26] to 10:1 [27]. We chose intermediate values of 140 integrase and 2000 nucleocapsid, which are consistent with recent reviews [28].

#### Nucleocapsid

Given the large number of nucleocapsid proteins and its small binding footprint, we did not model the steric properties of nucleocapsid explicitly during the condensation protocol. Instead, the RNA model was treated as an expanded worm-like chain with 1.5 nm radius, which was estimated from PDB entry 2l4l. A weak attractive component was applied to model interaction of the polycationic nucleocapsid with the polyanionic RNA. The direction of the displacement for bead i (Vi) is based on a distance-dependent summation of the vectors to all surrounding beads (Vij), excluding beads within a ±4 nt window in the chain around the bead:

$$Vi=\sum_{j}{Vij/r}_{ij}^{2}$$

The vector is then normalized and the magnitude of the displacement along this vector is constant over the simulation (Table 3).

We used a simple additive approach for the electrostatic nature of the interaction. If both beads have NC bound, the full attractive potential is applied; if one bead has NC bound, the potential is weighted by one half, and if both beads are free of NC, the potential is zero. We also tested two extreme assumptions for this potential, which gave the expected results. Full attraction for NC-NC and zero attraction for NC-RNA showed a strong exclusion of the 5’UTR similar to the “FullModel” results in Table 2, with the 5’UTR showing an average radius of 20.08 nm over 25 instances of Self-avoiding Gag model with integrase tetramers. Full attraction for both NC-NC and NC-RNA showed results similar to the “FullAllNC” control in Table 2, with the 5’UTR buried in the RNP with an average radius of 15.37 nm in Self-avoiding Gag models with integrase tetramers.

Nucleocapsid positions were treated explicitly, and were assigned in two steps. First, specific sites identified by CLIP-seq [9] were added, excluding positions that overlap with occupied integrase sites. These CLIP-seq sites were extracted from the data with a scanning window similarly to the specific integrase sites. Second, additional nucleocapsid sites were assigned randomly to fill the remaining portions of the two gRNA, with a minimum spacing of 3 beads. Nucleocapsid binds most tightly to single-stranded nucleic acids [17], so in our default models, random positions for NC were not assigned within the 5’UTR.

### Analysis methods

A lattice-based approximation of the convex hull was used to estimate the volume of RNP globules, and calculated similarly to previous work [14]. The average radial distance of all beads, or a selected subset of beads, relative to the center of gravity was used as a metric to quantify both compactness of a globule and asymmetry in placement of selected portions of the RNA chains. Contact probability plots were calculated by counting the number of contacts within a given threshold (here, 10 nm) within a given sequence bin, across all 25 instances of a particular model.

### Data sharing

Nine sets of models of the condensed HIV-1 RNP, using the three hypotheses for the starting model, and with integrase tetramers, dimers or no integrase, are available at https://zenodo.org/record/2662964. Open-source software and input data files for generating models with the self-avoiding Gag hypothesis are available on GitHub at https://github.com/dsgoodsell/HIVnucleoid.
